# Genomics against flatulence

**DOI:** 10.1111/j.1462-2920.2007.01400.x

**Published:** 2007-08

**Authors:** Michael Y Galperin

**Affiliations:** National Center for Biotechnology Information, National Library of Medicine, National Institutes of Health Bethesda, MD 20894, USA

In the past several months, eukaryotic genome sequencing has brought us the complete genome of the tiny green alga *Ostreococcus ‘lucimarinus’* (Palenik *et al*., 2007) and a draft genome of the mosquito *Aedes aegypti* (Nene *et al*., 2007). Given that the genome sequence of another *Ostreococcus* species, *Ostreococcus tauri*, has been sequenced less than a year ago (Derelle *et al*., 2006), the availability of the *O. lucimarinus* genome opens a possibility to study the evolution of this unicellular planktonic organism and its adaptation to the life in the surface layer of the sea. There has been also exciting news from archaeal and bacterial genome sequencing. The list of recently sequenced genomes (Table 1) includes bacteria that inhabit a variety of ecological niches and degrade numerous environmental contaminants, as well as two γ-proteobacteria with near-minimal gene sets.

**Table 1:** Recently completed microbial genomes (April–June 2007).

Species name	Taxonomy	GenBank accession	Genome size (bp)	Proteins (total)	Sequencing centre^a^	Reference
New organisms						
*Ostreococcus lucimarinus*	*Eukaryota, Viridiplantae*	CP000581–CP000601	13 200 000 (Total)	7651	JGI	Palenik *et al.* (2007)
*Methanobrevibacter smithii*	*Euryarchaeota*	CP000678	1 853 160	1795	Washington U.	Samuel *et al.* (2007)
*Metallosphaera sedula*	*Crenarchaeota*	CP000682	2 191 517	2256	JGI	Unpublished
*Pyrobaculum arsenaticum*	*Crenarchaeota*	CP000660	2 121 076	2298	JGI	Unpublished
*Clavibacter michiganensis*	*Actinobacteria*	AM711867AM711866AM711865	3 297 891 69 989 27 357	3029	University of Bielefeld, Germany	
*Mycobacterium gilvum*	*Actinobacteria*	CP000656–CP000659	5 982 829 (Total)	5579	JGI	Unpublished
*Salinispora tropica*	*Actinobacteria*	CP000667	5 183 331	4536	JGI	Udwary *et al*. (2007)
*Flavobacterium johnsoniae*	*Bacteroidetes*	CP000685	6 096 872	5017	JGI	Unpublished
*Prosthecochloris vibrioformis*	*Chlorobi*	CP000607	1 966 858	1753	JGI	Unpublished
*Dehalococcoides* sp. BAV1	*Chloroflexi*	CP000688	1 341 892	1371	JGI	Unpublished
*Roseiflexus* sp. RS-1	*Chloroflexi*	CP000686	5 801 598	4517	JGI	Unpublished
*Synechococcu*s sp. RCC307	*Cyanobacteria*	CT978603	2 224 914	2535	Genoscope	Unpublished
*Synechococcus* sp. WH 7803	*Cyanobacteria*	CT971583	2 366 980	2533	Genoscope	Unpublished
*Caldicellulosiruptor* *saccharolyticus*	*Firmicutes*	CP000679	2 970 275	2679	JGI	Unpublished
*Clostridium botulinum*	*Firmicutes*	AM412317AM412318	3 886 916 16 344	3592	Sanger Institute	Sebaihia *et al.* (2007)
*Lactobacillus reuteri*	*Firmicutes*	CP000705	1 999 618	1900	JGI	Unpublished
*Mycoplasma agalactiae*	*Firmicutes*	CU179680	877 438	742	Genoscope	Sirand-Pugnet *et al*. (2007)
*Pelotomaculum* *thermopropionicum*	*Firmicutes*	AP009389	3 025 375	2920	Marine Biotech. Institute	Kosaka *et al.* (2006)
*Streptococcus suis* 05ZYH33	*Firmicutes*	CP000407	2 096 309	2186	BIG – Beijing	Chen *et al.* (2007)
*Streptococcus suis* 98HAH33	*Firmicutes*	CP000408	2 095 698	2189	BIG – Beijing	Chen *et al.* (2007)
*Acidiphilium cryptum*	*α-Proteobacteria*	CP000689–CP000697	3 963 080 (Total)	3559	JGI	Unpublished
*Bradyrhizobium* sp. BTAi1	*α-Proteobacteria*	CP000494CP000495	8 264 687 228 826	7622	JGI	Giraud *et al*. (2007)
*Bradyrhizobium* sp. ORS278	*α-Proteobacteria*	CU234118	7 456 587	6717	Genoscope	Giraud *et al*. (2007)
*Brucella ovis*	*α-Proteobacteria*	CP000708CP000709	2 111 370 1 164 220	2892	TIGR	Unpublished
*Orientia tsutsugamushi*	*α-Proteobacteria*	AM494475	2 127 051	1182	Seoul National University, Korea	Cho *et al.* (2007)
*Sphingomonas wittichii*	*α-Proteobacteria*	CP000699CP000700CP000701	5 382 261 310 228 222 757	5345	JGI	Unpublished
*Polynucleobacter* sp. QLW-P1DMWA-1	*β-Proteobacteria*	CP000655	2 159 490	2077	JGI	Unpublished
*Aeromonas salmonicida*	*γ-Proteobacteria*	CP000644CP000645CP000646	4 702 402 166 749 155 098	4413	NRC – Halifax	Unpublished
Candidatus *Vesicomyosocius okutanii*	*γ-Proteobacteria*	AP009247	1 022 154	937	JAMSTEC	Kuwahara *et al.* (2007)
*Dichelobacter nodosus*	*γ-Proteobacteria*	CP000513	1 389 350	1280	TIGR	Myers *et al.* (2007)
*Enterobacter* sp. 638	*γ-Proteobacteria*	CP000653CP000654	4 518 712 157 749	4240	JGI	Unpublished
*Pseudomonas mendocina*	*γ-Proteobacteria*	CP000680	5 072 807	4594	JGI	Unpublished
*Pseudomonas stutzeri*	*γ-Proteobacteria*	CP000304	4 567 418	4128	BRI – Beijing	Unpublished
*Psychrobacter* sp. PRwf-1	*γ-Proteobacteria*	CP000713CP000714CP000715	2 978 976 13 956 2 117	2385	JGI	Unpublished
*Shewanella putrefaciens*	*γ-Proteobacteria*	CP000681	4 659 220	3972	JGI	Unpublished
*Geobacter uraniumreducens*	*δ-Proteobacteria*	CP000698	5 136 364	4357	JGI	Unpublished
*Thermotoga petrophila*	*Thermotogae*	CP000702	1 823 511	1785	JGI	Unpublished
New strains						
*Mycobacterium tuberculosis* H37Ra	*Actinobacteria*	CP000611	4 419 977	4034	Fudan University, Shanghai, China	Unpublished
*Staphylococcus aureus* spp. *aureus*JH9	*Firmicutes*	CP000703CP000704	2 906 700 30 429	2726	JGI	Unpublished
*Rhodobacter sphaeroides* ATCC 17025	*α-Proteobacteria*	CP000661–CP000666	4 557 127 (Total)	4333	JGI	Unpublished
*Legionella pneumophila* str. Corby	*γ-Proteobacteria*	CP000675	3 576 470	3206	Fritz Lipmann Institute, Jena, Germany	Unpublished
*Pseudomonas putida* F1	*γ-Proteobacteria*	CP000712	5 959 964	5252	JGI	Unpublished
*Vibrio cholerae*O395	*γ-Proteobacteria*	CP000627CP000626	3 024 069 1 108 250	3875	TIGR	Unpublished
*Yersinia pestis* Pestoides F	*γ-Proteobacteria*	CP000668CP000670CP000669	4 517 345 137 010 71 507	4069	JGI	Unpublished

a Sequencing centre names are abbreviated as follows: JGI, US Department of Energy Joint Genome Institute, Walnut Creek, CA, USA; Washington U., Washington University School of Medicine, St. Louis, MO, USA; Genoscope, Centre National de Séquençage, Evry cedex, France; Sanger Institute, The Wellcome Trust Sanger Institute, Hinxton, Cambridge, UK; Marine Biotech. Institute, Marine Biotechnology Institute, Heita, Kamaishi, Iwate, Japan; BIG – Beijing, Beijing Institute of Genomics, Chinese Academy of Sciences, Beijing, China; TIGR, The Institute of Genomic Research (since October 2006 – J. Craig Venter Institute), Rockville, MD, USA; NRC-Halifax, NRC Institute for Marine Biosciences, Halifax, Nova Scotia, Canada; JAMSTEC, Japan Agency for Maine-Earth Science and Technology, Natsushima-cho, Yokosuka, Japan; BRI – Beijing, The Biotechnology Research Institute, Chinese Academy of Agricultural Sciences, HaiDian, Beijing, China.

Archaea (archaebacteria) are generally viewed as free-living organisms capable of surviving at extremely high temperatures, salt concentrations and extreme pH values. The discoveries, primarily in the past several years, of various mesophilic archaea have done little to shatter the perception of archaea as exotic organisms with little relevance to everyday human life. Indeed, there are no known human pathogens among the *Archaea*. Nevertheless, archaea play a key role in human gut, accounting for one of its activities that usually goes unmentioned, namely, production of gas. Some of this gas, consisting largely of methane and hydrogen, makes it all the way back the gastrointestinal tract and shows up in human breath. Most of the gas, however, is released from the large intestine the natural way, by some estimates, up to 0.5 l day^−1^ (see McKay *et al*., 1985; Suarez *et al*., 1997). Although the excreted gas has a complex and variable chemical composition, its methane component is produced by methanogenic bacteria, which, as we now know, are actually not bacteria but archaea. Isolation of methanogens from human feces was first reported almost 40 years ago (Nottingham and Hungate, 1968). The isolated organism was similar to the ruminal methanogen *Methanobacterium ruminantium*, described by Paul H. Smith and Robert E. Hungate (1958) several years earlier. The culture counts, estimated from serial dilutions, were in the range of 2 × 10^7^ to 2 × 10^9^ organisms g^−1^. In the spirit of the time, the validity of the latter figure is discussed in the article (which is freely available on line at the *Journal of Bacteriology* website http://jb.asm.org/cgi/reprint/96/6/2178 or through PubMed Central) with the following details:

The sample showing 2 × 10^9^ methanogenic bacteria per gram was obtained from a patient in the Veterans Administration Hospital, Madison, Wis., by E. M. Lapinski. Unusually large quantities of methane had been detected in the expired gas of this patient. A fecal sample was sent airmail to Davis, California, sealed with no access of oxygen. Presumably, the lower than body temperature in transit prevented growth, and the value found is an approximation of the viable bacteria in the sample.

In a subsequent revision of metanogen taxonomy, *M. ruminantium* was transferred to the genus *Methanobrevibacter* with one of its species named *Methanobrevibacter smithii*, after Paul H. Smith (Balch *et al*., 1979). *Methanobrevibacter smithii* has been detected in human feces, human dental plaque and human vaginal samples, as well as in anaerobic environments such as activated sludge (Miller *et al*., 1982; Belay *et al*., 1988; 1990). The metabolic role of *M. smithii* and its interactions with bacterial gut flora have been subject of intensive studies (Samuel and Gordon, 2006), which recently culminated in determining the complete genome sequence of this organism. In the spirit of the current times, the description of *M. smithii* genome (Samuel *et al*., 2007) offers a detailed analysis of its intermediary metabolism but avoids mentioning the most conspicuous contribution of this organism to the human well-being. Still, the authors introduce the notion of ‘antiarchaeal’ drugs that could be used to control the population of *M. smithii* in the lower intestine. Taking into account the contribution of methanogenic archaea to the global warming (see our previous column, Galperin, 2007), a search for antimethanogen compounds could be quite an important undertaking. Accordingly, studies of methanogenesis and archaeal metabolism in general, which until recently appeared to be of purely academic interest, are suddenly finding unexpected applications in drug design.

Two other recently sequenced archaeal genomes belong to *Crenarchaea*. *Metallosphaera sedula* strain DSM 5348 is an aerobic thermoacidophile related to *Sulfolobus* spp. that was first isolated from a thermal pond in the Pisciarelli Solfatara in Italy (Huber *et al*., 1989). It can grow at temperatures ranging from 50 to 79°C with optimal growth at 74°C and pH 2. *Metallosphaera sedula* is capable of oxidizing sulfidic ores, such as pyrite, making it an attractive organism for use in bioleaching of metals. Several of its respiratory complexes have been characterized (Kappler *et al*., 2005); the genome sequence should allow identification of the rest of them.

Just 2 months after completing the sequence of *Pyrobaculum calidifontis* (GenBank accession number CP000561), JGI scientists have released the genome of *Pyrobaculum arsenaticum,* the fourth member of that genus with a completely sequenced genome. *Pyrobaculum arsenaticum* is a strictly anaerobic, hyperthermophilic archaeon, isolated from a hot spring in Italy. It could grow chemoautotrophically with CO_2_ as carbon source, H_2_ as electron donor and arsenate, thiosulfate or elemental sulfur as electron acceptors (Huber *et al*., 2000). It could also grow organotrophically, using sulfur, selenate or arsenate as electron acceptors. H_2_S was formed from sulfur or thiosulfate, arsenite from arsenate, whereas reduction of selenate produced elemental selenium. The variety of electron acceptors used by different *Pyrobaculum* spp. as well as their different tolerance towards oxygen make comparative analysis of these genomes a very interesting task.

Among all the members of the phylum *Actinobacteria* with completely sequenced genomes, *Clavibacter michiganensis* is only the second phytopathogen. The sequenced strain *C. michiganensis* spp. *michiganensis* is a pathogen of tomato that causes bacterial wilt and canker. This species includes four other subspecies. One of them, *C. michiganensis* spp. *sepedonicus*, is responsible for ring rot of potato; its genome is being sequenced at the Sanger Institute. Three other subspecies of *C. michiganensis* infect, respectively, maize, wheat and alfalfa (Jahr *et al*., 1999; Gartemann *et al*., 2003).

*Mycobacterium gilvum* (previously referred to as *Mycobacterium flavescens* strain PYR-GCK) has been isolated in the sediment from the Grand Calumet River in Indiana as a strain capable of using pyrene as a sole source of carbon and energy. This strain was also capable of metabolizing such polycyclic aromatic hydrocarbons (PAHs) as phenanthrene and fluoranthene, but not naphthalene, chrysene, anthracene, fluorene, or benzo[a]pyrene (Dean-Ross and Cerniglia, 1996). The first step of pyrene degradation is catalysed by the same two-subunit aromatic ring-hydroxylating dioxygenase NidAB as the one found in *Mycobacterium vanbaalenii* and other PAH-degrading mycobacteria (Brezna *et al*., 2003). Thus, *M. gilvum* probably differs from them in the downstream steps of PAH degradation, which now could be deduced through genome comparisons.

*Salinispora tropica* strain CNB-440 is a marine actinomycete, the first sequenced representative of the unique genus within the phylum *Actinobacteria* that is widespread in tropical and subtropical marine sediments (Jensen and Mafnas, 2006). Its cultivation has been achieved by including seawater into the growth medium (Mincer *et al*., 2002), which is why *Salinispora* spp. are now considered obligate marine bacteria. Accordingly, comparing the genome of *S. tropica* to other actinobacterial genomes offers a chance to examine how members of this genus have adapted to the marine life. JGI scientists are currently sequencing the genome of *Salinispora arenicola*, the second cultured representative of this genus (Maldonado *et al*., 2005). In addition to its ecological significance, *S. tropica* is remarkable as a producent of the various halogenated macrolides, including the anticancer agent salinosporamide A, a potent inhibitor of the 20S proteasome (Williams *et al*., 2005). A detailed analysis of *S. tropica* genome is expected to clarify the macrolide biosynthesis pathway(s) and allow genetic manipulation leading to an in improved production of salinosporamide A and other secondary metabolites (Udwary *et al*., 2007).

*Flavobacterium johnsoniae* (formerly *Cytophaga johnsonae*), first described by Roger Stanier (1947), is an aerobic bacterium that is commonly found in soil and freshwater. It is a member of the phylum *Bacteroidetes*, also known as the *Cytophaga-Flavobacterium-Bacteroides* group. *Flavobacterium johnsoniae* has attracted significant attention in the recent past, both as an effective chitin degrader and as a model organism for studying gliding motility (McBride, 2001; 2004). Remarkably, these two activities seem to be linked, as defects in gliding motility also affect chitin utilization (Braun *et al*., 2005). The reason for that is degradation of chitin and other insoluble biopolymers by *F. johnsoniae* apparently requires direct contact of cell with the substrate. This resembles cellulose digestion by the closely related *Cytophaga hutchinsonii*, whose genome has been sequenced at the JGI a year ago (Xie *et al*., 2007).

*Prosthecochloris vibrioformis* (a synonym of *Chlorobium phaeovibrioides*, Imhoff, 2003) is a green sulfur phototrophic bacterium, a member of the phylum *Chlorobi*, which already has four members with sequenced genomes. Like other *Chlorobi*, *P. vibrioformis* gains energy by anoxygenic photosynthesis using thiosulfate as an electron acceptor and reducing it to elemental sulfur, which accumulates as extracellular globules. The cells of *P. vibrioformis* have brownish colour owing to the large amounts of bacteriochlorophyll *e* and carotenoids in their chlorosomes.

*Roseiflexus* sp. strain RS-1, also an anoxygenic photosynthetic bacterium, is the first phototrophic representative of the phylum *Chloroflexi* to have a completely sequenced genome. The 5.2-Mb genome of another phototrophic member of the *Chloroflexi*, *Chloroflexus aurantiacus* strain J-10-fl has been available in GenBank for the past 5 years (accession no. AAAH00000000) but still remains at the stage of 77 contigs. Three more representatives of this phylum are members of the genus *Dehalococcoides* which have lost the photosynthetic genes in the process of genome contraction during their adaptation to reductive dehalogenation (see below). In contrast to *P. vibrioformis, Roseiflexus* sp. does not contain chlorosomes and its major photosynthetic pigment is bacteriochlorophyll a. Comparative analysis of the genomes of members of *Chlorobi* and *Chloroflexi* could provide valuable information about mechanisms of anoxygenic photosynthesis.

*Dehalococcoides* sp. strain BAV1 is also a member of *Chloroflexi*, the third representative of the genus *Dehalococcoides* to have a completely sequenced genome. All three *Dehalococcoides* spp. are capable of metabolizing chlorinated hydrocarbons, including tetrachloroethene (PCE) and trichloroethene (TCE), which are commonly used as solvents and are major contaminants of soil and groundwater. Although *Dehalococcoides ethenogenes* strain 195 was originally reported to be capable of metabolizing PCE and TCE all the way to ethene, its cultures were found to accumulate 1,1-dichloroethene and vinyl chloride, which is a known human carcinogen (Maymo-Gatell *et al*., 1997; 2001). *Dehalococcoides* sp. CBDB1 can dechlorinate a variety of chlorobenzens but is also incapable of using dichloroethene or vinyl chloride (Kube *et al*., 2005). In contrast, the newly sequenced *Dehalococcoides* sp. strain BAV1 could grow using vinyl chloride and all dichloroethene isomers as electron acceptors. In addition, it could cometabolize PCE and TCE. Thus, strain BAV1 can be used for complete detoxification of PCE and TCE, that is, degradation of these compounds to environmentally benign ethene and inorganic chloride (He *et al*., 2003a,b). Comparative analysis of all three strains could provide clues to the mechanisms of reductive dehalogenation and the substrate specificities of the corresponding enzymes.

*Clostridium botulinum* is a well-known agent of food poisoning. Its spores are resistant to heat and survive exposure to air. In anaerobic conditions, which often exist in poorly prepared canned foods, *C. botulinum* spores germinate into vegetative cells. Growing vegetative cells secrete a variety of proteases and one or more toxic neuropeptides, known collectively as the botulinum toxin. Consumption of food contaminated with nanogram quantities of the botulinum toxin can be fatal for humans, as the toxin causes paralysis of chest muscles, which leads to asphyxiation. In addition, *C. botulinum* occasionally infects open wounds. The genome description (Sebaihia *et al*., 2007) includes a detailed comparison of five *Clostridium* spp., particularly with respect to the structure of the cell surface, extracellular enzymes and the regulation of toxin production. One cannot help noting that this deadly toxin has become a popular tool in cosmetology and has been suggested for a number of other applications, such as treatment of chronic migraines.

Two other members of the order *Clostridiales* in the current list (Table 1) are finding more traditional uses in biotechnology. *Caldicellulosiruptor saccharolyticus* is an anaerobic thermophilic bacterium that was isolated from a thermal spring in New Zealand (Sissons *et al*., 1987). Both the genus name, meaning literally ‘hot cellulose disruptor’, and the species name, which means ‘lysing sugar’, reflect the ability of the organism to metabolize a variety of mono- and polysaccharides, such as arabinose, amorphous cellulose, fructose, galactose, glucose, glycogen, lactose, laminarin, lichenin, mannose, maltose, pullulan, pectin, rhamnose, starch, sucrose, xylan and xylose (Rainey *et al*., 1994). Based on its inability to form spores, *C. saccharolyticus* was first assigned to a separate lineage within the *Bacillus/Clostridium* subphylum of the Gram-positive bacteria (the current *Firmicutes*), but later recognized as a member of the order *Clostridiales*. The ability of *C. saccharolyticus* to grow at 70°C, hydrolysing both α- and β-glucans, including cellulose and pectin, makes it an attractive candidate for conversion of plant biomass into biofuel. Comparison of the genomic sequence of *C. saccharolyticus* with those of related organisms could provide an insight in the mechanisms and regulation of cellulose degradation.

*Pelotomaculum thermopropionicum*, also a member of *Clostridiales*, is a thermophilic propionate-oxidizing anaerobic bacterium, isolated in 2000 from anaerobic sludge blanket reactor in Niigata, Japan. It grows best in a syntrophic association with methanogenic archaea, metabolizing propionate, ethanol, lactate, butanol, pentanol, 1,3-propanediol, propanol and ethylene glycol (Imachi *et al*., 2002). Even before the completion of the genome sequencing, Kosaka and colleagues (2006) analysed the propionate fermentation pathway of *P. thermopropionicum* and compared it with the corresponding pathway of the actinobacterium *Propionibacterium acnes*.

*Acidiphilium cryptum* strain JF-5 has been isolated from an acidic coal mine lake situated in the Lusatian mining area in east central Germany. It is a facultatively anaerobic, extremely acidophilic heterotrophic α-proteobacterium that can reduce soluble and solid-phase Fe(III) using glucose as the electron donor (Küsel *et al*., 1999). It is also capable of reducing Cr(VI), which makes it an interesting model organism for studying metal reduction at extremely low pH values.

*Sphingomonas wittichii* (formerly *Sphingomonas* sp. strain RW1) is an aerobic α-proteobacterium that was isolated in the course of screening for dibenzo-*p*-dioxin-utilizing bacteria from the water samples collected from the River Elbe downstream of the city of Hamburg, Germany (Wittich *et al*., 1992). This organism could grow using both the biaryl ethers dibenzo-*p*-dioxin and dibenzofuran as the sole sources of carbon and energy. Polychlorinated dibenzo-*p*-dioxins and dibenzofurans are widespread contaminants of the environment that can be formed as by-products during the manufacture of pesticides, incineration of halogen-containing chemicals and of industrial and domestic waste, and bleaching of paper pulp (Wittich *et al*., 1992). The rare ability of *S. wittichii* to completely degrade these toxic compounds makes it an organism of choice for bioremediation of contaminated soil (Halden *et al*., 1999). While dioxin dioxygenase of *S. wittichii* has been characterized and the likely pathway of its degradation delineated several years ago (Armengaud *et al*., 1998; 1999), the genome sequence should lead to a much better understanding of the cellular adaptations to dioxin degradation.

Among the recently sequenced γ-proteobacterial genomes, two are remarkable because of their unusually small size. One of them, Candidatus *Vesicomyosocius okutanii* is an intracellular symbiont, inhabiting gill epithelial cells of the deep-sea clam *Calyptogena okutanii*s, which lives in the vicinity of hydrothermal vents (Kuwahara *et al*., 2007). In contrast to insect symbionts, some of which have very small genomes, Cand. *V. okutanii* is an autotroph, more specifically a thioautotroph. Its genome is also quite small, only 1 Mb in size, and codes for mere 937 proteins. Still, their products are sufficient to support thioautotrophic metabolism, as they include enzymes catalysing synthesis of almost all amino acids and various cofactors, RubisCO for CO_2_ fixation, as well as enzymes of glycolysis, pentose phosphate cycle, and partial TCA cycle. Sulfide is used as electron donor and converted to sulfate, while oxygen and nitrate serve as terminal electron acceptors. However, several supposedly essential genes are missing, e.g. cell division genes *ftsA*, *ftsL*, *ftsN*, *ftsX* and *ftsZ* and lipoprotein localization genes *lolA*, *lolB* and *lolC* (Kuwahara *et al*., 2007). The absence of cell division genes may be linked to the fact that each host cell carries only a single cell of Cand. *V. okutanii*, which is vertically transferred through clam oocytes.

If Cand. *V. okutanii* has the smallest autotrophic genome sequenced to date, the sheep pathogen *Dichelobacter nodosus* has the smallest genome of any anaerobe. The genome of *D. nodosus*, the causative agent of ovine foot rot, is less than 1.4 Mb in size and codes for less than 1300 proteins (Myers *et al*., 2007). However, these genes are apparently all significant, as there are few paralogues and very few pseudogenes. Given that *D. nodosus* belongs to an early branching group of *γ-Proteobacteria,* the authors suggest that its evolutionary history differed from that of most other organisms with very small genomes, which are either obligate intracellular pathogens or symbionts. Thus, massive gene loss through pseudogenization probably played only a minor role in extensive genome reduction, if any, in the evolution of *D. nodosus*.

Of the three recently sequenced pseudomonad genomes (Table 1), *Pseudomonas putida* F1 is by far the best-studied one. This obligately aerobic bacterium was originally isolated from a polluted creek in Urbana, Illinois, by enrichment with ethylbenzene as the sole source of carbon and energy (Gibson *et al*., 1968). This strain can also grow on benzene, toluene and *p*-cymene, which are common contaminants of groundwater, leaking from underground gasoline storage tanks. When provided with a carbon source for growth, *P. putida* F1 can oxidize a variety of aromatic and aliphatic compounds that do not support its growth. This list includes nitrotoluenes, chlorobenzenes, chlorophenols and trichloroethylene. The mechanism of toluene degradation by *P. putida* F1 toluene dioxygenase and its regulation have been studied in much detail (Zylstra *et al*., 1988; Lau *et al*., 1997). Remarkably, *P. putida* F1 senses benzene, ethylbenzene and trichloroethylene, perceiving them as chemoattractants (Parales *et al*., 2000). Both strains of *P. putida*, F1 and KT2240, whose genome was sequenced 5 years ago (Nelson *et al*., 2002; Dos Santos *et al*., 2004), have great potential for use in bioremediation.

The *Shewanella* genome sequencing project has released yet another complete genome, this time of the facultative anaerobe *Shewanella putrefaciens*. While some strains of *S. putrefaciens* have been isolated from clinical samples and appear to be opportunistic human pathogens (Holt *et al*., 2005), the sequenced strain is interesting primarily because of its capability to effectively reduce polyvalent metals and radionuclides including solid phase oxides of Fe, Mn, Cr, U(VI) and Tc (VII), using lactate as the electron donor.

The δ-proteobacterium *Geobacter uraniumreducens* is also a powerful reducer of metals. The sequenced strain *G. uraniumreducens* Rf4 has been isolated from a contaminated aquifer at the former uranium ore processing facility in Rifle, Colorado (Anderson *et al*., 2003). Injection of acetate (1–3 mM) into the groundwater led to an enrichment of the groundwater with *G. uraniumreducens*, which coincided with a decrease in U(VI) levels. Comparison of *G. uraniumreducens* genome sequence with that of *Geobacter metallireducens* and other metal-reducing bacteria should help in finding the best strains for bioremediation of uranium and other radionuclides.

Eight years after the publication of the genome sequence of an obligately anaerobic hyperthermophilic bacterium *Thermotoga maritima* (Nelson *et al*., 1999), the genome of a second member of the phylum *Thermotogae* has been released. While also an obligate anaerobe similar to *T. maritima* in its sugar utilization profile, *Thermotoga petrophila* strain RKU-1 has been isolated from a deep subterranean oil reservoir in Niigata, Japan, and could grow at a much wider range of temperatures, from 48 to 88°C, with an optimum at 80°C (Takahata *et al*., 2001). These features make T. petrophila an attractive source of thermostable enzymes, as well as a convenient model organism to study the mechanisms of thermotolerance in bacteria.

The list of the recently sequenced genomes also includes several important bacterial pathogens. The genome of *Orientia* (formerly *Rickettsia*) *tsutsugamushi*, the causative agent of scrub typhus, is remarkable primarily for the abundance of repeat elements, including numerous *tra* genes, transposases, phage integrases, reverse transcriptases and potential host–cell interaction proteins (Cho *et al*., 2007). Genome sequencing of two strains of *Streptococcus suis* serotype 2, which caused an outbreak of streptococcal toxic shock syndrome in China, revealed a shared pathogenicity island that appeared responsible for the high-virulence phenotype (Chen *et al*., 2007). In an article with an unusually impressive title, genome analysis of the ruminant pathogen *Mycoplasma agalactiae* and closely related mycoplasmas revealed traces of horizontal gene transfer, suggesting that it could have played a role in the mycoplasmal evolution (Sirand-Pugnet *et al*., 2007). The fifth complete genome of a *Brucella* spp. comes from the sheep pathogen *Brucella ovis*, which causes ovine contagious epididymitis in rams and premature abortion in pregnant ewes. It should be noted that in 1988, the ICSB Subcommittee on the Taxonomy of Brucella concluded that the *Brucella* is a monospecific genus with a single species *Brucella melitensis*. Indeed, all *Brucella* spp. have genomes consisting of two chromosomes with similar sizes, similar G + C content of 57.2% and encoding many proteins that are 99–100% identical. Thus, *B. ovis* is currently considered an accepted synonym for *Brucella melitensis* biovar Ovis.

The genome of *Aeromonas salmonicida* spp. salmonicida strain A449 was sequenced in an effort to find new ways to control this widespread fish pathogen. The draft version of the genome has already been used to construct *A. salmonicida* DNA microarray and identify potential virulence genes and candidates for vaccine development (Nash *et al*., 2006).

Last but not least, a recent article has uncovered the function of one of widespread ‘conserved hypothetical’ genes. This gene, which has been designated *yebR* in *Escherichia coli*, *ytsP* in *Bacillus subtilis* and YKL069w in *Saccharomyces cerevisiae*, has been shown to encode an enzyme that reduces free methionine sulfoxide (Lin *et al*., 2007). Two previously identified methionine sulfoxide reductases act predominantly on oxidized methionine residues in protein and cannot protect the cellular pool of free amino acid from oxidation. This work is yet another proof that ‘house-cleaning’ is a major cellular function that might employ a fair number of the uncharacterized ‘hypothetical’ proteins (see Galperin *et al*., 2006 for a review).
